# Novel *NHEJ1* pathogenic variant linked to severe combined immunodeficiency, microcephaly, and abnormal T and B cell receptor repertoires

**DOI:** 10.3389/fped.2022.883173

**Published:** 2022-07-27

**Authors:** Shirly Frizinsky, Erez Rechavi, Ortal Barel, Yu Nee Lee, Amos J. Simon, Atar Lev, Tali Stauber, Etai Adam, Raz Somech

**Affiliations:** ^1^Pediatric Department A and the Immunology Service, Jeffrey Modell Foundation Center, Edmond and Lily Safra Children’s Hospital, Sheba Medical Center, Ramat Gan, Israel; ^2^Sackler Faculty of Medicine, Tel Aviv University, Tel Aviv, Israel; ^3^The Wohl Institute for Translational Medicine and Cancer Research Center, Sheba Medical Center, Ramat Gan, Israel; ^4^Department of Pediatric Hematology, Oncology and Bone Marrow Transplant, Edmond and Lily Safra Children’s Hospital, Sheba Medical Center, Ramat Gan, Israel

**Keywords:** severe combined immunodeficiency (SCID), next-generation sequencing (NGS), TCR repertoire, *XLF/Cernunnos*, *NHEJ1*

## Abstract

**Background:**

During the process of generating diverse T and B cell receptor (TCR and BCR, respectively) repertoires, double-strand DNA breaks are produced. Subsequently, these breaks are corrected by a complex system led by the non-homologous end-joining (NHEJ). Pathogenic variants in genes involved in this process, such as the *NHEJ1* gene, cause severe combined immunodeficiency syndrome (SCID) along with neurodevelopmental disease and sensitivity to ionizing radiation.

**Objective:**

To provide new clinical and immunological insights on NHEJ1 deficiency arising from a newly diagnosed patient with severe immunodeficiency.

**Materials and methods:**

A male infant, born to consanguineous parents, suspected of having primary immunodeficiency underwent immunological and genetic workup. This included a thorough assessment of T cell phenotyping and lymphocyte activation by mitogen stimulation tests, whole-exome sequencing (WES), TCR repertoire Vβ repertoire *via* flow cytometry analysis, and TCR and BCR repertoire analysis *via* next-generation sequencing (NGS).

**Results:**

Clinical findings included microcephaly, recurrent pneumonia, and failure to thrive. An immune workup revealed lymphopenia, reduced T cell function, and hypogammaglobulinemia. Skewed TCR Vβ repertoire, TCR gamma (TRG) repertoire, and BCR repertoire were determined in the patient. Genetic analysis identified a novel homozygous missense pathogenic variant in *XLF/Cernunnos*: c.A580Ins.T; p.M194fs. The patient underwent a successful hematopoietic stem cell transplantation (HSCT).

**Conclusion:**

A novel *NHEJ1* pathogenic variant is reported in a patient who presented with SCID phenotype that displayed clonally expanded T and B cells. An adjusted HSCT was safe to ensure full T cell immune reconstitution.

## Introduction

The ability of the adaptive immune system to produce a polyclonal diverse repertoire of antigen receptors is critical for its function. This is obtained through T and B cell maturation process that includes the recombination of V(D)J gene segments (1). This process starts with double-strand DNA breaks (DSBs) and ends with new coding joints, and the development of new specific antigen receptors. Many proteins take part in this cascade, among them are the recombination-activating gene (RAG)-1 and -2 proteins which act during the initial stage when DSBs are formed (2). The repair of the damaged DSBs is performed using 2 mechanisms: the non-homologous end-joining (NHEJ) and the homologous recombination repair (HRR). NHEJ, the major pathway, includes DNA-end binding complex Ku70-Ku80, DNA-PKcs, Artemis, DNA Ligase IV, and XLF (XRCC4 like factor), also called Cernunnos proteins (3–5). During the NHEJ pathway, NHEJ1 interacts with XRCC4-Ligase IV, thereby repairing the broken DNA (6, 7). Genetic defects in proteins involved in the V(D)J recombination process can cause severe combined immunodeficiency (SCID). Such SCID is usually characterized by T cell-negative (T-), B cell-negative (B-), and natural killer cell-positive (NK+) immune phenotype (8–11). SCID can be categorized as a typical SCID or, if less severe, leaky SCID based upon the severity of T cell qualitative and quantitative deficiency (12). Patients with SCID require a hematopoietic stem cell transplantation (HSCT) from an optimal matched donor, but haplo-donors may also be used with successful results. So far, only a few cases of SCID due to NHEJ pathway pathogenic variants, and specifically defects in NHEJ1 protein, have been reported (13–18). These patients were characterized by recurrent infections, growth retardation, microcephaly, and few autoimmune manifestations. The ability of such patients to produce T and B cell receptor (TCR, BCR) repertoire has rarely been investigated (19, 20).

Herein we describe a patient harboring a novel *NHEJ1* pathogenic variant who underwent successful HSCT, yet with engraftment of low but selective amounts of donor’s cells. We further extend the knowledge about the immunodeficiency associated with pathogenic variant in *NHEJ1* by deciphering the patient’s TCR and B BCR repertoire using next-generation sequencing (NGS).

## Materials and methods

### Clinical data

Patient information was obtained from the electronic medical record of our hospital. The guardians were interviewed, and the patient was examined by the authors. Informed written consent was obtained, and all procedures were performed in accordance with the ethical standards of the institutional and/or national research committee and with the Helsinki declaration.

### Immune function

Cell surface markers of peripheral blood mononuclear cells (PBMCs) were determined by immunofluorescent staining using flow cytometry (FACS, NAVIOS, Beckman Coulter) with antibodies purchased from Beckman Coulter. Lymphocyte proliferation was done in response to Phytohemagglutinin and anti-CD3 (using tritiated thymidine incorporation). The cells were harvested 3 days after collection, and samples were counted on a liquid scintillation counter. All assays were performed in triplicate, and a stimulation index was calculated as the ratio between stimulated and unstimulated lymphocyte responses. The resultant stimulation index was compared with the stimulation index obtained from normal controls. Serum concentration of immunoglobulins was measured by nephelometry.

### Quantification of T cell receptor excision circles

The T cell receptor excision circle (TREC) analysis was performed using DNA extracted from the study patients’ PBMCs. The amount of signal joint TREC copies per DNA content was determined by real-time quantitative PCR (RQ-PCR). Reactions were performed using 0.5-mcg of genomic DNA and PCRs contained TaqMan universal PCR master mix (Applied Biosystems, Waltham, MA, United States), specific primers (900 nM), and FAM-TAMRA probes (250 nM). RQ-PCR was carried out in step one plus (Applied Biosystems, Waltham, MA, United States). The number of TRECs in a given sample was estimated by comparing the cycle threshold value obtained with a standard curve obtained from PCRs performed with 10-fold serial dilutions of an internal standard. Amplification of RNAseP (Taq-Man assay, Applied Biosystems, Waltham, MA, United States) served as a quality control to verify similar amounts of genomic DNA that were used in the assays.

### T cell receptor repertoire

Representatives of specific TCR-Variable β families were detected and quantified using the patient’s PBMCs with flow cytometry (NAVIOS, Beckman Coulter, Inc, Brea, California, United States) according to the manufacturer’s instructions (Beta Mark TCR Vβ repertoire kit, Beckman Coulter, Inc, Brea, California, United States). Normal control values comprised of 58 healthy people were obtained from the kit.

### Next-generation sequencing

T cell receptor and IGH libraries were generated from patient and control genomic DNA using primers for conserved regions of V and J genes in the TCR-gamma (*TRG*) locus and IGH, respectively, according to the manufacturer’s protocol (Lymphotrack; Invivoscibe Technologies^®^, Carlsbad, CA, United States). Quantified libraries were pooled and sequenced using Mi-Seq Illumia technology^®^. FASTA files from the filtered sequences were submitted to the ImMunoGeneTics (IMGT) HighV-QUEST webserver^®^,^1^ filtered for productive sequences only (no stop codons or frame shifts) and analyzed (21). Analyses were performed on CDR3 amino acid sequences. For TCR repertoires, V and J gene usage patterns were analyzed. Repertoire diversity was calculated using Shannon’s and Gini-Simpson’s diversity indices (22).

### Exome sequencing analysis and Sanger sequencing

High throughput sequencing for whole-exome sequencing was performed on genomic DNA patient samples. Coding regions were enriched with a SureSelect Human All Exon V5 Kit (Agilent, Santa Clara, United States) and then sequenced as 100-bp paired-end runs on an Illumina HiSeq 2500 (Illumina Inc., San Diego, CA, United States). We used the BWA mem algorithm (version 0.7.12) (23) for the alignment of the sequence reads to the human reference genome (hg19).

The HaplotypeCaller algorithm of GATK version 3.4 was applied for variant calling, as recommended in the best practice pipeline (24). KGG-seq v.08 was used for annotation of identified variants (25) and in-house scripts were applied for filtering, based on family pedigree and a local dataset of variants detected in previous sequencing projects. The *NHEJ1* pathogenic variant was validated by dideoxy Sanger sequencing in the patients and carriers. Data were evaluated using the Sequencer v5.0 software (Gene Codes Corporation, Ann Arbor, MI, United States).

## Results

### Clinical description

The patient was born prematurely, at 34 + 5 weeks of gestation. His parents were healthy and consanguineous (first-degree cousins). He had a clinically healthy 3-year-old brother. Since birth, the patient suffered recurrent skin rash and, at 4 months, began to have frequent episodes of pneumonia. Preliminary evaluation demonstrated lymphopenia and hypogammaglobulinemia and therefore he was referred to our hospital for further evaluation. Preliminary examination revealed extreme cachexia, tachypnea, and dyspnea, with clubbing of his fingernails. He had dysmorphic features with microcephaly (head circumference below third percentile), triangle shaped face, and developmental delay. A diffused maculopapular rash was seen with a suspected fungal origin. Initial inquiry revealed bilateral pneumonia with sustained *Respiratory syncytial virus* infection, and bacterial pneumonia due to *Klebsiella Pneumonia* infection. Further lymphoproliferative, metabolic, and autoimmune investigations were all normal.

### Immunological evaluation

Immunologic investigation revealed lymphopenia, low IgG levels, and with normal IgA and IgM levels (Table 1). Lymphocyte immuno-phenotyping showed abnormal representation for T, B, and NK cells, with a significant deficiency of CD3^+^ and CD20^+^ cells, and an increased percentage of CD56^+^ cells, consistent with a T^–^ B^–^ NK^+^ SCID phenotype. Lymphocyte proliferation was markedly reduced following stimulation with Phytohemagglutinin (PHA) or anti CD3. Thymus activity, determined by the quantification of TRECs was absent (Table 1). Although this was consistent with SCID, the patient still had a few residual T cell that we attempted to characterize. Analysis of the TCR repertoire was performed using both flow-cytometry analysis and NGS.

**TABLE 1:** Immune workup.

Status at presentation	Pre-HCST	Post-HCST	Normal range*
**CBC (Cells/ml × 10^–3^)**			
WBC	3.9	4.9	5.2–11
Monocytes	0.4	0.65	0.2–1
Lymphocytes	1.27	1.8	1.5–6.5
Hemoglobin (g/dl)	11.8	12.4	11–14
Platelets	308	180	150–400
Eosinophils	0.02	0.07	0–0.7
**Lymphocyte subsets [Cells/mm3 (%)]**			
Lymphocytes	0.643 (22.4)	1.617	2.3–5.4
T (CD3^+^)	0.135 (21)	1.035	1.4–3.7 (60–85)
T helper (CD4^+^)	0.148 (23)	0.437	0.7–2.2 (36–63)
T cytotoxic (CD8^+^)	0.148 (23)	0.647	0.49–1.3 (15–40)
B (CD 20^+^)	0.019	0.065	0.05–0.3
NK (CD16+CD56+) (%)	70	30	6–30
**Serum immunoglobulin (mg/dl)**			
IgA	41	<26	38–222
IgM	119	27.7	56–208
IgG	376	462	590–1430
IgE	<4.8	Not available	0–90
TRECs (Copies per 0.5 μg DNA)	0	5280	>400
TCR	Restricted	Polyclonal	

Pre- and post-hematopoietic stem cell transplantation (HSCT) immune workup in the reported patient with non-homologous end-joining-1 (NHEJ1) pathogenic variant.

*Healthy donors, aged 1–2 years, with percentages/counts presented as median (10th and 90th percentiles) (48). HSCT, hematopoietic stem cell transplantation; TRECs, T-cell receptor excision circles; TCR, T-cell receptor repertoire.

**FIGURE 1:**
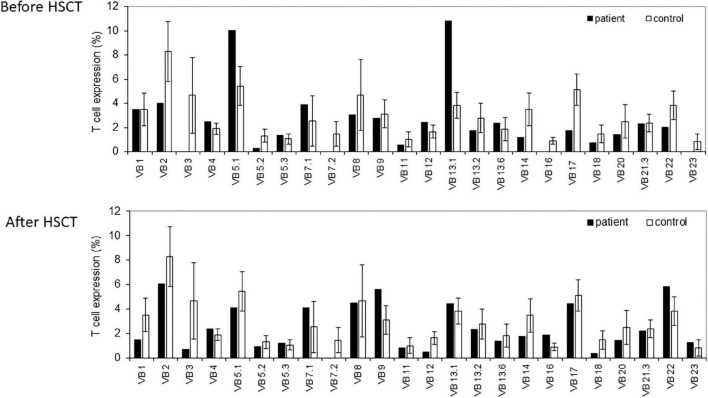
T cell receptor (TCR) Vβ repertoire analyses. Flow cytometry analysis of surface membrane expression of 24 T cell receptor β chain’s variable gene families, in our non-homologous end-joining-1 (*NHEJ1*) deficient patients (black bars), compared with healthy controls (white bars), before (upper panel), and after (lower panel) the hematopoietic stem cell transplantation (HSCT).

**FIGURE 2:**
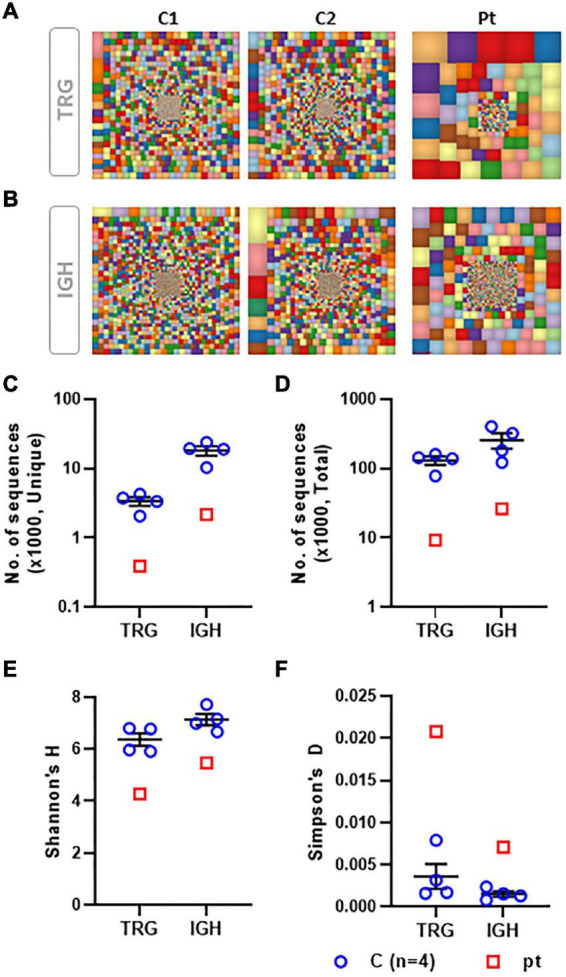
Characteristics of the TRG and IGH repertoire determined by NGS. Graphical presentation of the TCR gamma (TRG) repertoire using the Tree map program where each square represents a specific clone and the size of the square represent the frequency of the clone, for TRG **(A)** and IGH **(B)** repertoires. A scatter dot plot presenting the unique **(C)** and total **(D)** number of sequences for TRG and IGH repertoires. A scatter dot plot presenting the Shannon’s H diversity index **(E)** and the Simpson’s Index of Unevenness **(F)** for TRG and IGH repertoires. TRG and IGH repertoire analysis from a total of four controls were compared with our patient’s data.

The TCR repertoire was assessed with the TCR-Vβ assay using flow cytometry. This resulted in a skewed repertoire with two clonally expanded TCRs (vβ 5.1 and vβ 13.1) and underrepresentation of 10 different TCRs (Figure 1). High-throughput immune sequencing of the TRG chain repertoire was performed on the patient’s PBMCs, and a healthy age-matched control. The patient expressed a restricted repertoire with clonal expansion cells as can be observed by the Tree-maps, which represent the TRG repertoire (Figure 2A). Similar restriction and clonal expansion was observed in the patient’s Treemaps in the IGH, representing the BCR repertoire (Figure 2B). This is further observed in the unique and total number of sequence, which were about 10-fold less in the patient compared with controls for both TRG and IGH repertoires (Figures 2C,D). The Shannon’s H diversity index, which accounts for both the total number of sequences and the overall level of clonal expansions, was lower for the patient compared with controls (Figure 2E). The Simpson’s D index, which measures the unevenness of the repertoire, showed unevenness in the patient’s TRG and IGH repertoires, compared with controls (Figure 2F). These abnormal TCR and IGH repertoires indicate a possible defect in the V(D)J gene recombination process and/or abnormal T and B cell selections within the thymus or bone marrow, respectively.

### Exome sequencing analysis and Sanger sequencing

Due to familial consanguinity, a homozygous recessive disease trait was suspected. Genetic analysis by whole exome sequencing identified a novel bi-allelic homozygous frame shift pathogenic variant in the *NHEJ1* gene (*XLF/Cernunnos*) (c.A580Ins.T; p.M194fs). This was confirmed by direct Sanger sequencing. Segregation of the identified pathogenic variant with the disease phenotype, was confirmed within the patient’s family with both parents being carriers for the pathogenic variant and a healthy brother who harbors a wild-type *NHEJ1* gene. The WES did not reveal any other pathogenic variant relevant to immunodeficiency or immune-dysregulation. The *NHEJ1* novel variant was not found in our in-house exomes (*n* = 1,500), and was not present with a minor allele frequency (MAF) ≥ 0.01 in either the 1,000 Genomes Project (1KG) or dbSNP 135 database or the NHLBI Exome Sequencing Project (ESP^2^). This *NHEJ1* pathogenic variant is rare and different from previously described patients (17). Our patient shared several clinical and laboratory characteristics described in previous human cases (13–16) and animal model (26–28), thereby supporting the hypothesis that this *NHEJ1* variant is a disease-causing pathogenic variant.

### Clinical outcome

At the age of 14 months, the patient underwent HSCT from a matched related donor. The underlying genetic defect required a special conditioning protocol with reduced-intensity protocol, based on the 2021 ESID guidelines for HSCT for inborn errors of immunity (29). This included cyclophosphamide (cumulative dose 20 mg/kg), fludarabine (cumulative dose 150 mg/m^2^), and Grafalon anti-thymocyte globulin (ATG) (cumulative dose 20 mg/kg). The conditioning regimen was a slightly modified version of protocol F in the aforementioned guidelines, as follows:

Fludarabine 30 mg/m^2^/dose daily on days (−9) to (−5).

ATG Grafalon 5 mg/kg/dose daily on days (−9) to (−6).

Cyclophosphamide 5 mg/kg/dose daily on days (−5) to (−2).

A bone marrow graft contained total nucleated cell count of 3 × 10^8^/kg of recipient, CD34^+^ 10 × 10^6^/kg of recipient, and CD3^+^ 0.3 × 10^8^/kg of recipient. Graft vs. host (GVHD) prophylaxis consisted of cyclosporine and mycophenolate mofetil.

During the post-transplantation period, the patient had mild skin GVHD (treated with oral steroids) chronic lung disease, feeding difficulties, and failure to thrive, which required nasogastric feeding tube. Gastrointestinal GVHD was ruled out *via* gastric and rectal endoscopy and biopsy. The patient was hospitalized for a total of 4.5 months post-HSCT, during which there was a clinically and laboratory improvements. He had begun gaining weight, he was free from severe infections and was discharged with continued anti-bacterial and anti-viral prophylaxis. Anti-Bacillus Calmette–Guérin (anti-BCG) therapy was also given due to past vaccination. By 6 months of post-HSCT, he had normal CD4+, CD8+, CD20+, and CD56+ counts on flow cytometry of his peripheral blood (Table 1).

The functional assay for evaluating T cell immune reconstitution using the repeated measurements of TREC level, as well as the presentation of his TCR repertoire were normal, demonstrated significant improvement (Figure 1).

Despite his good clinical and laboratory condition, microsatellites analysis on peripheral blood lymphocytes post-transplantation revealed low level and mixed chimerism status, ranging from 30 to 50% of donor cells. Further investigation by preforming sorted T cells microsatellites evaluation, 3 months post-HSCT, resulted in a 100% donor cells, suggesting full recovery of his T cell compartment. This was consistent with his good clinical condition and blood work. Immunosuppression therapy was gradually decreased, and he was discharged on the 130-day post-transplantation with prophylactic antibiotics, anti-viral, and anti-mycobacterial treatment. During his post-transplantation follow-up period, he was clinically well and after signs of B cell engraftment, was weaned off of prophylactic treatment and immunoglobulins replacement therapy. At the last clinical visit, at the age of 2 years, the patient was doing well and free of infections.

## Discussion

Here, we report a patient with a clinical and immunological phenotype of SCID. The patient harbored a novel homozygous pathogenic variant in *NHEJ1*, a part of the NHEJ complex. We characterized his TCR and BCR repertoires using flow cytometry and NGS to demonstrate the effect of the *NHEJ1* pathogenic variant on the ability to produce normal diverse TCR and BCR repertories. The patient underwent a successful HSCT, with full engraftment of T cells that were detected only by a specific split chimerism test.

Pathogenic variants in the NHEJ complex cause T and B lymphocytes maturation arrest, sensitivity to IR, and a clinical and laboratory T^–^ B^–^ NK^+^ SCID phenotype (11, 13, 30–33). Pathogenic variants in *DCLRE1C (Artemis)* (11) and *XRCC4/Ligase IV* (33, 34) that may also include microcephaly and developmental delay where described in association with leukemia and lymphoma (35, 36). In 2006, the role of a newly identified protein, XLF/Cernunnos, was elucidated (13). Since then, several studies deciphered XLF function (37, 38), and it is now known that this protein is a key player during DNA repair. Its interaction with XRCC4-Ligase IV is a crucial step. Another important role is the contribution to junctional diversity during V(D)J recombination, by stimulating N-nucleotide insertion (17). This may be responsible for an abnormal TCR diversity. Biallelic pathogenic variants in *NHEJ1* have been previously described, first in 2006 (13) with the description of five patients who presented with severe combined immunodeficiency. These patients shared clinical features of microcephaly and growth retardation. They demonstrated an immune phenotype of T^–^ B^–^ NK^+^ SCID and *in vitro* sensitivity to IR. As opposed to other forms of SCID, few patients were diagnosed at a fairly older age, some as teenagers, most probably due to a leaky pathogenic variant or partial function of residual cells. Since then more than a dozen patients with *NHEJ1* biallelic pathogenic variants were described (14–18). These patients shared similar clinical manifestations of susceptibility to opportunistic infections, microcephaly [except for one patient (14)], and growth retardation. Furthermore, autoimmunity was described (13–16, 18). Patients presented later in life, and survived several years despite infections otherwise lethal in other forms of SCID. Except for one case (18), all patients demonstrated an immunological profile consistent with of T^–^ B^–^ NK^+^ SCID. The former had normal B cell count and normal IgM and IgA levels, suggesting a leaky form of this deficiency. Further immune workup in several patients (17) revealed skewed TCR repertoire and reduced N-nucleotides insertion during V(D)J recombination. Of what is known so far regarding the aforementioned patients, 3 patients died from sepsis and 17 survived, among them, 11 who received HSCT (17). Due to the small number of patients, an accurate genotype/phenotype correlation for this disease cannot be established yet.

Pathogenic variants in *NHEJ1* result mainly in extreme sensitivity to IR, genome instability, failure to thrive, and microcephaly. The effect on immunity is perhaps milder (13, 14) than other forms of SCID. This discrepancy was demonstrated *in vitro* in a mice model (35) where pathogenic variant in *NHEJ1* caused only a mild defect in lymphocyte maturation, although cells were severely impaired in their ability to support V(D)J recombination. The study suggested a compensatory mechanism during V(D)J recombination. This might explain why many patients present later in life and survive the risk of severe infections. Yet, a curative treatment may still be HSCT. In 2018, the outcome of HSCT in PID patients with DSB repair disorders, was published (36). Of 87 patients, 17 were genetically diagnosed with *NHEJ1* pathogenic variant. About 69% of patients who received conditioning survived, with better survival rates in those receiving reduced-intensity protocol, perhaps due to DNA instability and radio sensitivity, as previously discussed. A review on the use of HSCT in patients with PID that was published in 2019 (39), conclude that the decision-making on HSCT should be done after taking in consideration of the specific diagnosis and indication, timing of the procedure, and perhaps most importantly, the risks of HSCT against the risks of disease progression.

Herein we describe a newly diagnosed patient carrying a novel homozygous pathogenic variant in *XLF/Cernunnos*. The infant shared clinical and immunological features with previously reported cases, suggestive of SCID, with lymphocyte maturation arrest and developmental delay.

The early diagnosis and treatment in cases of SCID, is of immense importance. In several countries, such as Israel and the United States, newborn screening (NBS) is used for different diseases among them, SCID. NBS for SCID includes the use of TREC quantification in Guthrie cards. This highly specific and sensitive assay was proven to be effective in the early diagnosis and prompt treatment of affected infants. Patients with typical SCID (infants with less than 300 T-cells), are identified by the screening. Moreover, patients with leaky SCID (e.g., Omenn syndrome) and some patients with reduce or dysfunctional T cells can be identified by the NBS. Additionally, syndromic patients (e.g., 22q11.2 deletion syndrome or Trisomy 21), or newborns with secondary T cell lymphopenia (e.g., chylothorax), or severely premature infants are sometimes found to be positive in the screening. Lastly, some patients with idiopathic T cell lymphopenia are identified as well. This group of patients desires a close follow-up to determine the nature of their diseases. Our patient did not undergo NBS (due to his place of origin, where NBS is not available), and was diagnosed while clinically symptomatic. As in previous cases, the patient presented increased susceptibility to viral and bacterial infections, microcephaly, and failure to thrive. Further immunologic profile analysis demonstrated impaired cellular and humoral immunity feature common to previously described patients.

Profiling the patient’s TCR and BCR repertoires, using NGS, revealed a skewed repertoire (19, 20). Although the latter may be attributed to secondary environmental triggers (e.g., infections) that drive clonally expanded cells, they reflect the underlying genetic defect. The recent development of NGS techniques enabled analysis of immune repertoires to a depth that was unreached previously. This can be used in different clinical setups, such as immunodeficiencies (40–42), autoimmune disorders (43), and infections (44). The advantage of the NGS technology is that it ensures the finding of the clonal receptor rearrangements in every patient due to the enormous depth of sequencing. It allows for the detection of multiple sub-clones, specific preferential usage of V, D, and J gene segments, and complementarity determining region 3 (CDR3) characteristics and to look for clonotypic sharing in patients with a similar disease (45). Altogether, these collective data can be used to diagnose immunodeficiency and appreciate more precisely its depth, to monitor the response to treatment, and for the early identification of disease exacerbation and even to correlate the immune repertoire with specific mutations. In the case of post-bone marrow transplantation, the assessment of the reconstitution of the cell receptor repertoire is essential in predicting outcome.

Our approach to define underlying genetic mutations is to use whole-exome sequencing. The debate regarding choosing one approach (whole-exome sequencing) over another (targeted gene panel sequencing) is extensively discussed in the literature with known advantages and disadvantages for each. Compared with targeted panels, whole-exome sequencing has the advantages of reduced cost, simplified workflow, and the potential for the identification of novel disease-related genes (46). Nevertheless, during the interpretation of the exome sequencing data, we suggest prioritizing specific genes that are related to primary immunodeficiency, and only if negative to look for novel genetic mutations (47).

As with many previously reported patients (14–18), our patient underwent successful HSCT from a matched related donor (sibling). A close follow-up demonstrated cellular immune reconstitution with normal TCR (Table 1), and normal lymphocyte proliferation. It is important to achieve high amount of donor cells, but simple detection in the peripheral blood is not always sufficient, and a better assessment of the engraftment quality mandates cell split tests.

Non-homologous end-joining defects are associated with neurodevelopmental problems and IR susceptibility (17). Our patient presented with microcephaly and neurodevelopmental delay. Obviously, these factors will not be corrected by HSCT, which only replaces the factors of immunity, but does not change the course of the neurological disease, nor reduce the IR sensitivity. A continued follow-up and monitoring is required.

In summary, we presented a patient with a novel *NHEJ1* pathogenic variant who displayed typical features. HSCT cured his immune symptoms including the abnormal TCR repertoire. This was in correlation to a full T cells engraftment, as seen by a specific split chimerism assay. The latter is an important and informative test that should be used in special cases to verify the success of the HSCT procedure.

## Data availability statement

The data sets presented in this article are not readily available, since parental consent was not obtained for sharing the raw data with public databases. Requests to access the datasets should be directed to the corresponding author.

## Ethics statement

The studies involving human participants were reviewed and approved by Institutional Review Board, Sheba Medical Center. Written informed consent to participate in this study was provided by the participants’ legal guardian/next of kin.

## Author contributions

SF, ER, TS, and RS initiated the study, obtained clinical data and analyzed it, and wrote the manuscript and critically reviewed it. EA obtained clinical data and analyzed it. YL, AS, and AL performed and analyzed the immunological studies. OB interpreted the exome sequencing data. All authors critically reviewed the manuscript and approved the submitted version.

## Footnotes

1
http://www.imgt.org

2
http://evs.gs.washington.edu/EVS/
